# Mouse dendritic cells and other myeloid subtypes in healthy lymph nodes and skin: 26‐Color flow cytometry panel for immune phenotyping

**DOI:** 10.1002/eji.202250004

**Published:** 2022-08-25

**Authors:** Florian Hornsteiner, Martina M Sykora, Christoph H Tripp, Sieghart Sopper, Patrizia Stoitzner

**Affiliations:** ^1^ Department of Dermatology, Venereology & Allergology Medical University of Innsbruck Innsbruck Austria; ^2^ Internal Medicine V, Hematology and Oncology Medical University of Innsbruck Innsbruck Austria; ^3^ Tyrolean Cancer Research Center Innsbruck Austria; ^4^ Department Internal Medicine I University Hospital Tübingen Tübingen Germany

**Keywords:** myeloid cells, dendritic cells, immune phenotyping, mouse lymph node, mouse skin

## Abstract

This novel 26‐color flow cytometry panel allows the detailed immune phenotyping of the complex network of myeloid cells in murine lymph nodes and skin. With the optimized panel the different murine DC‐subsets and other myeloid cell types can be identified and further characterized for co‐stimulatory and inhibitory surface molecules.

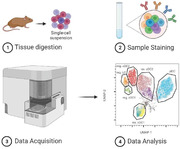

We here describe the establishment and optimization of a 26‐color flow cytometry panel to dissect the complex network of myeloid cells in mouse lymphoid and non‐lymphoid tissue with a specific focus on dendritic cells (DC). This multicolor panel allows a detailed separation of the different DC‐subsets, namely conventional DC type 1 (cDC1), cDC2, Langerhans cells (LC), and plasmacytoid DC (pDC) with simultaneous identification of NK cells, T cells, B cells as well as monocytes, neutrophils and macrophages. Due to inclusion of additional markers characterizing DC, the migratory status and expression of co‐stimulatory and inhibitory molecules can be determined on the various DC‐subsets.

As key regulators of the immune system DC contribute to a broad spectrum of innate and adaptive immune responses against pathogens, allergens, and cancer [1]. In the peripheral tissue, DC encounter pathogens and migrate to the draining lymph nodes (LN), where they instruct T cells to home to tissue for clearance of pathogens [2]. The skin contains a complex network of DC with LC and dermal DC which can be subdivided into XCR1^+^ cDC1, CD11b^+^ cDC2, and CD11b^–^ cDC2 [3]. As defining the myeloid subsets remains challenging [4], the aim was to establish a novel multicolor flow cytometry panel to discriminate DC from other myeloid cells. Based on markers that were previously described in the literature [3, 4, 5, 6] and with our own DC‐expertise we designed this 26‐color flow cytometry panel (Fig. S1, Table S1). Here, we report the development and validation of this novel DC‐panel in healthy mouse LN and skin.

This panel was optimized using the Cytek^®^ Aurora, a full spectrum cytometer equipped with five lasers and 64 detectors (Table S2) [7]. The materials and methods including antibody list (Table S3), single color reference controls (Table S4), titration process (Fig. S2), and panel development and optimization (Tables S5 and S6, Figs. S3‐S5) are highlighted in the Supporting Information.

A representative gating tree of the 26‐color staining on skin‐draining LN is shown in Figure 1. After several preclearance steps, dead cells were excluded from the analysis using the fixable viability dye eF780 (Fig. 1A; Fig. S6). The gating was performed according to fluorescence minus one (FMO) controls (Fig. S7). First, CD19^+^ B cells, NK1.1^+^ NK cells as well as CD4^+^ and CD8^+^ T cells were identified in CD45^+^ immune cells (Fig. 1B). Next, pDC were gated by pDCA‐1 and monocytes (Ly‐6C^+^Ly‐6G^–^) as well as neutrophils (Ly‐6C^+^Ly‐6G^+^) were characterized. Furthermore, CD11b^+^CD11c^–^ cells were excluded from the final in‐depth DC‐analysis (Fig. 1C).

**Figure 1:**
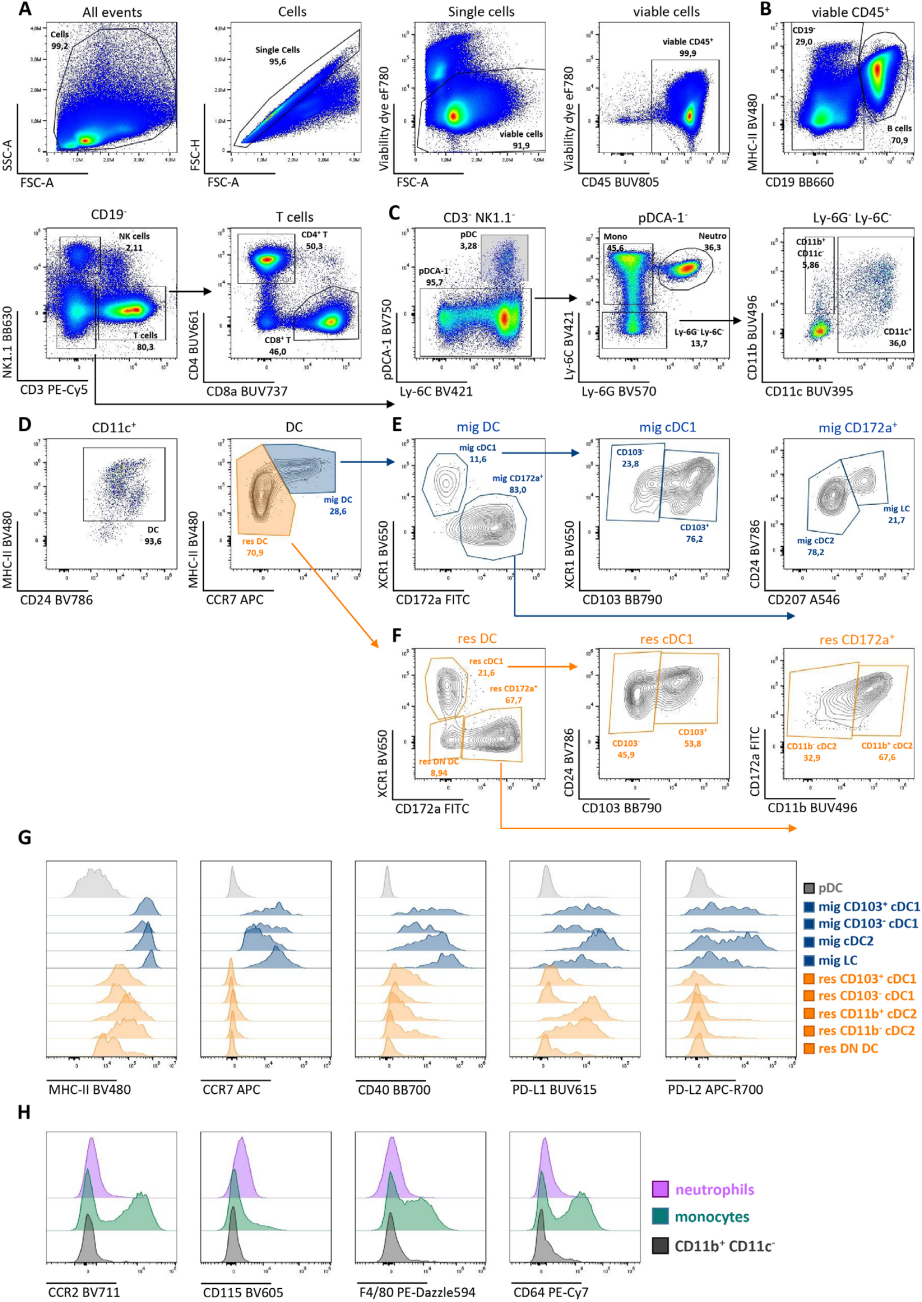
Gating strategy for mouse skin‐draining LN. Inguinal LN were enzymatically digested to generate single cell suspensions. (A) Gating strategy for viable CD45^+^ cells after exclusion of cellular debris, doublets, and dead cells. (B) CD19^+^MHC‐II^+^ B cells, NK1.1^+^ NK cells, and CD3^+^ T cells were identified with further separation into CD4^+^ and CD8^+^ T cell subsets. (C) From non‐lymphoid cells, pDCA‐1^+^Ly‐6C^+^ pDC, Ly‐6C^+^Ly‐6G^–^ monocytes, and Ly‐6C^+^Ly‐6G^+^ neutrophils were discriminated. CD11b versus CD11c gating separated DC from non‐DC myeloid cells. D. CD11c^+^ population contains MHC‐II^+^ DC that were subdivided into MHC‐II^high^CCR7^+^ migratory DC and MHC‐II^+^CCR7^–^ resident DC. (E) Migratory DC consist of XCR1^+^CD172a^–^ cDC1 with CD103^–^ and CD103^+^ subpopulations, CD24^–^CD207^–^ cDC2, and CD24^+^CD207^+^ LC. (F) Resident DC were divided into XCR1 expressing CD103^–^ and CD103^+^ cDC1. The cDC2 were separated into CD172a^–^XCR1^–^ DN DC and CD11b^–^ and CD11b^+^ cDC2. (G) Expression of phenotypical markers on DC‐subsets: pDC (grey), migratory DC‐subsets (blue), and resident DC‐subsets (orange). (H) Expression of markers on non‐DC myeloid cells (neutrophils, monocytes, and CD11b^+^CD11c^–^ cells). Mono…monocytes, Neutro…neutrophils, mig DC…migratory DC, res DC…resident DC, DN‐DC…double‐negative DC. One representative example is shown for three mice analyzed in three separate experiments

We proceeded with gating CD11c^+^MHC‐II^+^ cells, a commonly used strategy for DC in LN (Fig. 1D). The chemokine receptor CCR7 allows to distinguish migratory (CD11c^+^MHC‐II^high^CCR7^+^) and resident (CD11c^+^MHC‐II^+^CCR7^–^) DC (Fig. 1D) [8]. Representative histograms demonstrate that migratory DC‐subsets display higher levels of MHC‐II and CCR7 compared to resident DC‐subsets (Fig. 1G).

Defining the different DC‐subsets is challenging as they share the expression of several markers. LC and cDC1 both express MHC‐II, CD11c, CD24, and CD207 despite their different localization, ontogeny, lifespan, and functional properties [3]. Thus, we divided migratory and resident DC subsets into XCR1 and CD172a expressing cells. XCR1‐positive cDC1 contain CD103^+^ and CD103^–^ subsets (Fig. 1 E‐F) [9, 10]. Resident DC also contain double‐negative (DN) (XCR1^–^CD172a^–^) cells which are poorly defined (Fig. 1F) [11]. The CD172a^+^ migratory DC consist of cDC2 and LC, which can be separated into CD207^+^CD24^+^ LC and cDC2 (Fig. 1E). Resident cDC2 (XCR1^–^CD172a^+^) can be further subdivided into CD11b^–^ and CD11b^+^ subsets (Fig. 1F). DC are characterized by a high functional plasticity, as they express co‐stimulatory molecules, i.e. CD40 and CD86, but also inhibitory ligands, like PD‐L1 and PD‐L2. These markers allow detailed phenotypical characterization of DC‐subsets (Fig. 1G).

Moreover, we investigated expression of CCR2, CD115, F4/80, and CD64 on different non‐DC myeloid cell populations, such as Ly6C^+^Ly6G^–^ monocytes, Ly6C^+^Ly6G^+^ neutrophils, and CD11b^+^CD11c^–^ cells. The monocyte population contains a CCR2^+^F4/80^+^CD64^+^ subset most likely recruited monocytes with very few CD11c^+^ monocyte‐derived DC (Fig. 1H, Fig. S8) [12]. The CD11b^+^CD11c^–^ population is CCR2^–^CD64^–^F4/80^–^ indicative that they are resident macrophages (Fig. 1H). We also performed computational analysis of the LN DC‐subsets with uniform manifold approximation projection (UMAP) analysis as shown in Figure S9.

Our novel multi‐color flow cytometry panel was also tested on mouse skin. After excluding cellular debris, doublets, and dead cells, we gated with the help of FMO controls (Fig. S10) on viable CD45^+^ cells (Fig. 2A). In healthy skin, the CD3^+^ T cell population consists of CD3^int^CD4^+^ T cells and CD3^high^γδ T cells, whereas CD8^+^ T cells, NK cells, B cells, neutrophils and pDC are rare in healthy skin (Fig. 2B). By following gating published earlier [13], we identified several myeloid non‐DC populations (P1‐P5) in the dermis (Fig. 2C) and different DC‐subsets (Fig. 2D, E). From the non CD11b^+^CD24^–^ population we identified CD11c^+^MHC‐II^+^ DC. Next, we divided DC into XCR1^+^ cDC1, which contain CD103^+^ and CD103^–^ subsets, and CD172a^+^ DC which consists mainly of CD207^+^ LC (Fig. 2D). Dermal cDC2 are characterized from the CD64^–^Ly‐6C^–^ population by gating on CD11c^+^MHC‐II^+^ cells that express CD11b and CD172a (Fig. 2E). In addition, we investigated CD115, F4/80 and CD64 expression on the non‐DC myeloid cell populations P1‐P5 (Fig. 2F). Analysis of CD40, PD‐L1, PD‐L2 and CCR7 on the different DC‐subets allows further phenotypical characterization (Fig. 2G). An example for UMAP analysis is shown in Figure S11 confirming manual gating for DC‐subsets and non‐DC populations.

**Figure 2:**
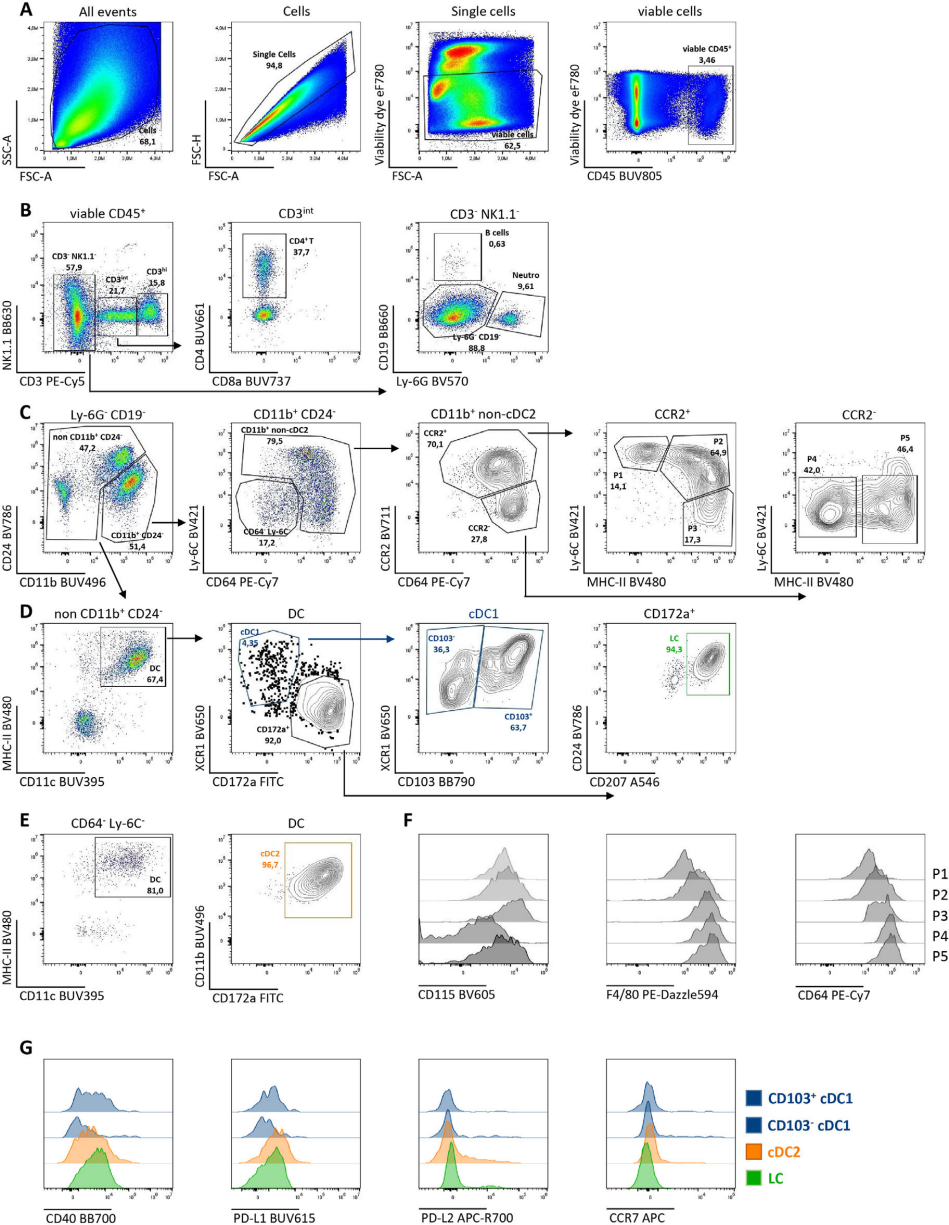
Gating strategy for mouse ear skin. Ear skin was enzymatically digested to obtain single cell suspensions. (A) Gating strategy for viable CD45^+^ cells after exclusion of cellular debris, doublets, and dead cells. (B) CD3^int^CD4^+^ T cells were identified, while NK1.1^+^ NK cells, CD8^+^ T cells, CD19^+^ B cells, and Ly‐6G^+^neutrophils are absent from healthy skin. (C) CD11b^+^CD24^–^ contain dermal monocytes, monocyte‐derived DC, and macrophages. Ly‐6C^high^MHC‐II^–^ monocytes (P1), Ly‐6C^high^MHC‐II^+^ (P2), and Ly‐6C^–^MHC‐II^+^ (P3) monocyte‐derived DC were identified among CD64^+^CCR2^+^ cells. CD64^+^CCR2^–^ cells consist of Ly‐6C^–^MHC‐II^–^ (P4) and Ly‐6C^–^ MHC‐II^+^ macrophages (P5). (D) CD11b^+^CD24^–^ population contains CD11c^+^MHC‐II^+^ DC, which were subdivided into XCR1^+^ cDC1 with CD103^+/‐^ subpopulations and CD172a^+^CD207^+^CD24^+^ LC. E. CD64^–^Ly‐6C^–^ cells mainly consist of CD11c^+^MHC‐II^+^ DC containing CD11b^+^CD172a^+^ cDC2. (F) Expression of markers on non‐DC myeloid dermal cell subsets P1 to P5. (G) Expression of markers on DC‐subsets: cDC1 (blue), cDC2 (orange), and LC (green). CD3^int^…CD3‐intermediate, CD3^hi^…CD3‐high, Neutro…neutrophils. One representative example is shown for three mice analyzed in three separate experiments

In summary, this novel optimized 26‐color flow cytometry panel allows the phenotyping of myeloid subsets with a special focus on DC in healthy mouse LN and skin. In addition to resolving the complexity of myeloid cells, this panel also provides basic information on the main lymphoid subsets.

## Conflict of Interest

The authors declare no commercial or financial conflict of interest.

### Peer review

The peer review history for this article is available at https://publons.com/publon/10.1002/eji.202250004

**Abbreviations**
DC: dendritic cells
LN: lymph nodes
cDC: conventional dendritic cells
pDC: plasmacytoid dendritic cells
LC: Langerhans cells
FMO: Fluorescence minus one

## Supporting information

Supplementary Figure S1: Myeloid cell subset delineation.
Supplementary Table 1. Markers for identification of myeloid cells
Supplementary Table 3: Antibody list
Supplementary Table 4: List of single stains used for unmixing of fluorochromes
Supplementary Figure S2. Antibody titrations for panel optimization
Supplementary Table 5: Panel Iterations
Supplementary Table 6: Explanations for panel iterations
Supplementary Figure S3: Comparison of CCR7 staining in lymph node cells for panel optimization.
Supplementary Figure S4: Comparison of CCR2 staining in lymph node cells for panel optimization.
Supplementary Figure S5: Comparison of different antibodies and clones to gate LC for panel optimization.
Supplementary Figure S6. Several preclearance steps were performed.
Supplementary Figure S7: Selected fluorescent minus one (FMO) staining controls for optimizing the panel for skin‐draining lymph node cells.
Supplementary Figure S8: Analysis of Ly‐6C^+^ monocytes
Supplementary Figure S9: UMAP analysis of the final 26‐color flow cytometry panel on mouse skin‐draining lymph node cells.
Supplementary Figure S10: Selected fluorescent minus one (FMO) staining controls for optimizing the panel for mouse ear skin.
Supplementary Figure S11: UMAP analysis of the final 26‐color flow cytometry panel on mouse ear skin.

## Data Availability

The flow cytometry files are available from the corresponding author upon reasonable request.
