# Supplementary material for: Mouse dendritic cells and other myeloid subtypes in healthy lymph nodes and skin: 26‐Color flow cytometry panel for immune phenotyping
Source: Eur J Immunol. 2022 Aug 25;52(12):2006–9. doi: 10.1002/eji.202250004 (PMC10087122; doi:10.1002/eji.202250004)
Supplement: Supplementary file 1 — Supplementary Figure S1: Myeloid cell subset delineation. Supplementary Table 1. Markers for identification of myeloid cells Supplementary Table 3: Antibody list Supplementary Table 4: List of single stains used for unmixing of fluorochromes Supplementary Figure S2. Antibody titrations for panel optimization Supplementary Table 5: Panel Iterations Supplementary Table 6: Explanations for panel iterations Supplementary Figure S3: Comparison of CCR7 staining in lymph node cells for panel optimization. Supplementary Figure S4: Comparison of CCR2 staining in lymph node cells for panel optimization. Supplementary Figure S5: Comparison of different antibodies and clones to gate LC for panel optimization. Supplementary Figure S6. Several preclearance steps were performed. Supplementary Figure S7: Selected fluorescent minus one (FMO) staining controls for optimizing the panel for skin‐draining lymph node cells. Supplementary Figure S8: Analysis of Ly‐6C+ monocytes Supplementary Figure S9: UMAP analysis of the final 26‐color flow cytometry panel on mouse skin‐draining lymph node cells. Supplementary Figure S10: Selected fluorescent minus one (FMO) staining controls for optimizing the panel for mouse ear skin. Supplementary Figure S11: UMAP analysis of the final 26‐color flow cytometry panel on mouse ear skin. [file EJI-52-2006-s001.docx]

**Supplementary Information**

**Supplementary Figure 1**


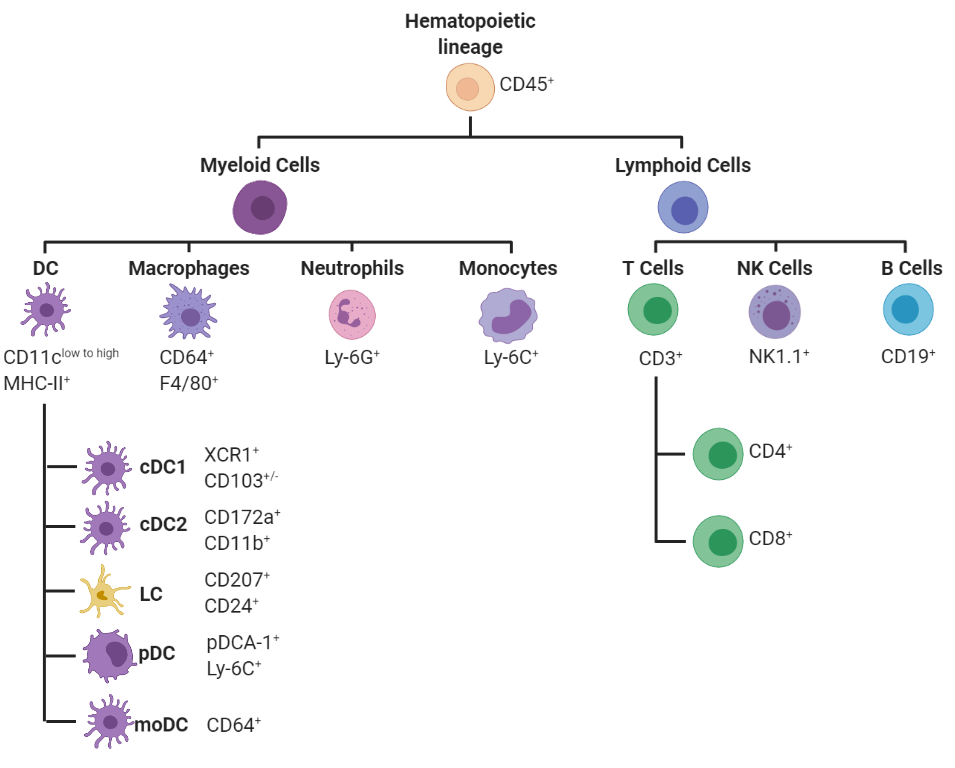


**Supplementary Figure S1: Myeloid cell subset delineation.** The in silico planning of the 26-marker myeloid panel involved identification of markers by our own DC-expertise and markers described in the literature to discriminate myeloid cell populations [1, 2]. Created with BioRender.com.

**Supplementary Table 1. Markers for identification of myeloid cells**

| Specificity | Alternative Name | Clone | Fluorochrome | Purpose |
| --- | --- | --- | --- | --- |
| CD172a | SIRPα | P84 | FITC | DC phenotyping |
| NK1.1 |  | PK136 | BB630 | NK cells |
| CD19 |  | 1D3 | BB660 | B cells |
| CD40 |  | 3/23 | BB700 | co-stimulatory molecule |
| CD103 | ITGAE, Integrin alpha-E | M290 | BB790 | DC phenotyping |
|  |  |  |  |  |
| CD207 | Langerin | 929F3.01 | A546 | LC |
| F4/80 |  | BM8 | PE-Dazzle594 | macrophages, LC |
| CD3e |  | 145-2C11 | PE-Cy5 | T cells |
| CD64 | FcγRI | X54 | PE-Cy7 | monocytes |
|  |  |  |  |  |
| CD197 | Chemokine receptor 7 (CCR7) | 4B12 | APC | migratory DC |
| CD273 | PD-L2 | TY25 | APC-R700 | co-inhibitory molecule |
| Viability dye eF780 |  |  | APC-Cy7 | viability |
|  |  |  |  |  |
| Ly-6C |  | HK1.4 | BV421 | monocytes |
| I-A/I-E | MHC class II | M5/114 | BV480 | DC lineage marker |
| Ly-6G |  | 1A8 | BV570 | neutrophils |
| CD115 | M-CSF-R | T38-320 | BV605 | myeloid cell phenotyping |
| XCR1 |  | ZET | BV650 | DC phenotyping |
| CD192 | Chemokine receptor 2 (CCR2) | 475301 | BV711 | migratory monocytes |
| CD317 | pDCA-1, Bst2 | 927 | BV750 | plasmacytoid DC |
| CD24 |  | M1/69 | BV786 | DC phenotyping |
|  |  |  |  |  |
| CD11c | ITGAX, Integrin alpha-X | N418 | BUV395 | DC lineage marker |
| CD11b | ITGAM, Integrin alpha-M | M1/70 | BUV496 | DC phenotyping |
| CD274 | PD-L1 | MIH5 | BUV615 | co-inhibitory molecule |
| CD4 |  | RM4-5 | BUV661 | CD4 T cells |
| CD8a |  | 53-6.7 | BUV737 | CD8 T cells |
| CD45 | Protein tyrosine phosphatase, receptor type, C | 30-F11 | BUV805 | hematopoetic cell lineage |

**Supplementary Table 2:**

This panel was optimized using the Cytek® Aurora, a full spectrum cytometer equipped with 5 lasers and 64 detectors. We took advantage that full spectrum cytometry allows the measurement of the entire emission spectra for each fluorochrome across all lasers in comparison to classical flow cytometry, which measures the peak emission of fluorochromes [3].

**Aurora Configuration.** 16 detectors of the UV laser (355 nm), 16 detectors of the violet laser (405 nm), 14 detectors of the blue laser (488 nm), 10 detectors of the yellow green laser (561 nm) and 8 detectors of the red laser (640 nm). Optical filter specification for each channel are listed as center wavelength and bandwidth. Fluorochromes used in this panel are assigned to their peak intensity channel.

| **Wavelength  (nm)** | **Laser power (mW)** | **Channel** | **Center Wavelength (nm)** | **Bandwidth  (nm)** | **Fluorochrome  (Peak intensity)** |
| --- | --- | --- | --- | --- | --- |
|  | | | | | |
| **UV laser** | | | | | |
| **355** | **20** | **UV1** | **373** | **15** |  |
|  | | **UV2** | **388** | **15** | **BUV395** |
|  |  | **UV3** | **428** | **15** |  |
|  |  | **UV4** | **443** | **15** |  |
|  |  | **UV5** | **458** | **15** |  |
|  |  | **UV6** | **473** | **15** |  |
|  |  | **UV7** | **514** | **28** | **BUV496** |
|  |  | **UV8** | **542** | **28** |  |
|  |  | **UV9** | **582** | **31** |  |
|  |  | **UV10** | **613** | **31** | **BUV615** |
|  |  | **UV11** | **664** | **27** | **BUV661** |
|  |  | **UV12** | **692** | **28** |  |
|  |  | **UV13** | **720** | **29** |  |
|  |  | **UV14** | **750** | **30** | **BUV737** |
|  |  | **UV15** | **780** | **30** |  |
|  |  | **UV16** | **812** | **34** | **BUV805** |
|  | | | | | |
| **Violet laser** | | | | | |
| **405** | **100** | **V1** | **428** | **15** | **BV421** |
|  | | **V2** | **443** | **15** |  |
|  |  | **V3** | **458** | **15** |  |
|  |  | **V4** | **473** | **15** |  |
|  |  | **V5** | **508** | **20** | **BV480** |
|  |  | **V6** | **525** | **17** |  |
|  |  | **V7** | **542** | **17** |  |
|  |  | **V8** | **581** | **19** | **BV570** |
|  |  | **V9** | **598** | **20** |  |
|  |  | **V10** | **615** | **20** | **BV605** |
|  |  | **V11** | **664** | **27** | **BV650** |
|  |  | **V12** | **692** | **28** |  |
|  |  | **V13** | **720** | **29** | **BV711** |
|  |  | **V14** | **750** | **30** | **BV750** |
|  |  | **V15** | **780** | **30** | **BV786** |
|  |  | **V16** | **812** | **34** |  |

| **Wavelength (nM)** | **Laser power (mW)** | **Channel** | **Center Wavelength (nm)** | **Bandwidth (nM)** | **Fluorochrome  (Peak intensity)** |
| --- | --- | --- | --- | --- | --- |
|  | | | | | |
| **Blue laser** | | | | | |
| **488** | **50** | **B1** | **508** | **20** |  |
|  | | **B2** | **525** | **17** | **FITC** |
|  |  | **B3** | **542** | **17** |  |
|  |  | **B4** | **581** | **19** |  |
|  |  | **B5** | **598** | **20** |  |
|  |  | **B6** | **615** | **20** | **BB630** |
|  |  | **B7** | **661** | **17** | **BB660** |
|  |  | **B8** | **679** | **18** |  |
|  |  | **B9** | **697** | **19** | **BB700** |
|  |  | **B10** | **717** | **20** |  |
|  |  | **B11** | **738** | **21** |  |
|  |  | **B12** | **760** | **23** |  |
|  |  | **B13** | **783** | **23** |  |
|  |  | **B14** | **812** | **34** | **BB790** |
|  | | | | | |
| **Yellow Green laser** | | | | | |
| **561** | **50** | **YG1** | **577** | **20** | **PE** |
|  | | **YG2** | **598** | **20** |  |
|  |  | **YG3** | **615** | **20** | **PE-Dazzle594** |
|  |  | **YG4** | **661** | **17** |  |
|  |  | **YG5** | **679** | **18** | **PE-Cy5** |
|  |  | **YG6** | **697** | **19** |  |
|  |  | **YG7** | **720** | **29** |  |
|  |  | **YG8** | **750** | **30** | **PE-Cy7** |
|  |  | **YG9** | **780** | **30** |  |
|  |  | **YG10** | **812** | **34** |  |
|  | | | | | |
| **Red laser** | | | | | |
| **640** | **80** | **R1** | **661** | **17** | **APC** |
|  | | **R2** | **679** | **18** |  |
|  |  | **R3** | **697** | **19** |  |
|  |  | **R4** | **717** | **20** | **APC-R700** |
|  |  | **R5** | **738** | **21** |  |
|  |  | **R6** | **760** | **23** |  |
|  |  | **R7** | **783** | **23** | **eFluor 780** |
|  |  | **R8** | **812** | **34** |  |

**Supplementary Materials and Methods:**

In this part, we describe the materials and methods used to prepare single cell suspensions from lymph nodes, spleen (used for titrations and cell compensation) and ear skin. Moreover, we differentiated bone-marrow derived DC (BMDC) and used day 6 BMDC for compensation purpose.

**Material:**

*Commercially available reagents:*

- PBS (Gibco)
- Heat inactivated Fetal Calf Serum (FCS) (PAN Biotech)
- Collagenase D (Roche)
- DNase I (Roche)
- Liberase (Roche)
- EDTA (Lonza)
- Hank’s Salt Solution with Ca^2+^ and Mg^2+^ (Biochrom)
- Hank’s Salt Solution w/o Ca^2+^ and Mg^2+^ (Biochrom)
- Bovine Serum Albumin (BSA, SERVA)
- Deionized water (DI H_2_0)
- RPMI 1640 (PAN Biotech: w/o L-glutamine, with 2.0 g/L NaHCO_3_
- Gentamicin (Gibco)
- Recombinant mouse GM-CSF (Biolegend)
- Brilliant Staining buffer (BD #563794)
- eFluor-780 fixable viability dye (eF780) (eBioscience #65-0865-14)
- Fc-Block – purified anti-CD16/CD32 antibody (clone 2.4G2) (TONBO Bioscience #70-0161)
- VersaComp Antibody Capture Kit (Beckman Coulter Life Sciences #B22804)
- Fixation/Permeabilization Solution Kit (BD #554714)
- Perm/Wash buffer (BD #554723)

*Buffers:*

Hank’s Salt Solution for digestion of lymph nodes and spleen:

Hank’s Salt Solution w/o Ca^2+^ and w/o Mg^2+^ supplemented with 2% of heat-inactivated FCS

Erylsis buffer (10X): 🡪 adjust to pH 7.3, for use dilute with DI H_2_0 to 1X

- 250 ml Aqua dest.
- 22.48 g NH_4_Cl
- 2.5 g KHCO_3_
- 93 mg EDTA

R10 culture medium: RPMI 1640 supplemented with 10% heat inactivated FCS, 2 mM L-glutamine and 50 µg/ml gentamycin

Staining buffer: PBS supplemented with 1 % BSA, 50 µM EDTA and 50 µg/ml DNAse I

Live/Dead dye solution: dilute eFluor-780 fixable viability dye 1:5000 in PBS

Brilliant Staining buffer: Mix 1 part of Staining buffer with 1 part of Brilliant Staining buffer

Perm/Wash buffer 1X: dilute 10X Perm/Wash buffer in DI H_2_0 to make a 1X solution prior to use

**Supplementary Table 3: Antibody list**

| Antigen | Fluorochrome | Clone | Manufacturer | Catalog # | Dilution |
| --- | --- | --- | --- | --- | --- |
| CD172a | FITC | P84 | Biolegend | 144005 | 1:100 |
| NK1.1 | BB630 | PK136 | BD | Custom-made | 1:200 |
| CD19 | BB660 | 1D3 | BD | Custom-made | 1:400 |
| CD40 | BB700 | 3/23 | BD | 742136 | 1:100 |
| CD103 | BB790 | M290 | BD | Custom-made | 1:200 |
|  |  |  |  |  |  |
| CD207 | A546 | 929F3.01 | Dendritics | DDX0362 | 1:100 |
| F4/80 | PE-Dazzle594 | BM8 | Biolegend | 123146 | 1:100 |
| CD3e | PE-Cy5 | 145-2C11 | BD | 553065 | 1:200 |
| CD64 | PE-Cy7 | X54 | Biolegend | 139314 | 1:200 |
|  |  |  |  |  |  |
| CCR7 | APC | 4B12 | Biolegend | 120108 | 1:50 |
| PD-L2 | APC-R700 | TY25 | BD | Custom-made | 1:100 |
| Viability dye eF780 | APC-Cy7 | - | eBioscience | 65-0865-14 | 1:5000 |
|  |  |  |  |  |  |
| Ly-6C | BV421 | HK1.4 | Biolegend | 128031 | 1:200 |
| MHC class II | BV480 | M5/114 | BD | 566086 | 1:400 |
| Ly-6G | BV570 | 1A8 | BD | Custom-made | 1:200 |
| CD115 | BV605 | T38-320 | BD | 743640 | 1:100 |
| XCR1 | BV650 | ZET | Biolegend | 148220 | 1:400 |
| CCR2 | BV711 | 475301 | BD | 747964 | 1:400 |
| pDCA-1 | BV750 | 927 | BD | 747608 | 1:400 |
| CD24 | BV786 | M1/69 | BD | 744470 | 1:400 |
|  |  |  |  |  |  |
| CD11c | BUV395 | N418 | BD | 744180 | 1:100 |
| CD11b | BUV496 | M1/70 | BD | Custom-made | 1:400 |
| PD-L1 | BUV615 | MIH5 | BD | Custom-made | 1:400 |
| CD4 | BUV661 | RM4-5 | BD | 741461 | 1:400 |
| CD8a | BUV737 | 53-6.7 | BD | 612759 | 1:400 |
| CD45 | BUV805 | 30-F11 | BD | 748370 | 1:200 |

**Methods:**

*Lymph node and spleen digestion protocol:*

1. Dissect skin-draining lymph nodes or spleen (for titration and cell compensation) from 6-8 week old C57BL/6 mice and transfer them into petri dish with 5 ml Hank’s Salt Solution (w/o Mg^2+^, Ca^2+^) supplemented with 2% FCS
2. Tear tissue apart and transfer into a 50 ml conical tube
3. Wash petri dish with 5 ml Hank’s Salt Solution (w/o Mg^2+^, Ca^2+^) supplemented with 2% FCS and transfer solution into the same 50 ml conical tube
4. For tissue digestion add 250 µg/ml Collagenase D and 300 µg/ml DNase I
5. Digest for 25 minutes at 37°C shaking in a water bath
6. Stop digestion with 5 mM EDTA
7. Press tissue pieces through a 100 µm cell strainer into a new 50 ml conical tube to obtain single-cell suspensions and wash with Staining buffer
8. Centrifuge at 485 x *g* for 5 minutes at 4°C
9. For spleen, erylysis is required. Resuspend splenocytes in 10 ml of 1X Erylysis buffer and incubate for 3 minutes at RT. Wash with Staining buffer and centrifuge at 485 x *g* for 5 minutes at 4°C
10. Resuspend cells in Staining buffer and filter through a 40 µm cell strainer
11. Count cells

*Ear skin digestion protocol:*

1. Use mouse ears from 6-8 week old C57BL/6 mice and transfer them into a 24-well-plate with 2 ml Hank’s Salt Solution with Ca^2+^ and Mg^2+^
2. Separate the ventral and the dorsal side of the ears and thoroughly cut them into small pieces
3. For tissue digestion add 150 µg/ml Liberase and 120 µg/ml DNase I
4. Digest for 45 minutes at 37°C
5. Stop digestion with 5 mM EDTA
6. Use a 5 ml syringe to pipette the cell suspension up and down for disruption of tissue
7. press tissue through a 100 µm cell strainer into a 50 ml conical tube to obtain single-cell suspensions and wash with Staining buffer
8. Centrifuge at 485 x *g* for 5 minutes at 4°C
9. Resuspend cells in Staining buffer and filter through a 40 µm cell strainer
10. Count cells

*Bone-marrow derived DC differentiation protocol:*

1. Carefully prepare tibia and femur with scissors without breaking the bone and place bones into a petri dish with 10 ml R10 medium
2. Transfer bones to petri dish with 70% ethanol to disinfect bones for 3 minutes
3. Transfer bones into a fresh petri dish with 10 ml R10 medium
4. Cut bones near the joints with the scissor
5. Flush out bone marrow with R10 medium by using a 1 ml syringe
6. Pipette bone marrow up and down to dissolve aggregates with a 5 ml pipette
7. Filter cell suspension through a 100 µm cell strainer into a 50 ml conical tube, wash petri dish with 10 ml R 10 medium and cell strainer with another 10 ml R10 medium
8. Centrifuge at 485 x *g* for 5 minutes at 4°C
9. For red blood cell lysis, resuspend cells in 10 ml of 1X Erylysis buffer and incubate for 3 minutes at RT. Complete with R10 medium and centrifuge at 485 x *g* for 5 minutes at 4°C
10. Resuspend cells in 5 ml of R10 medium and filter through a 40 µm cell strainer
11. Count cells

*BMDC-culture in 6-well-plates*

**Day 0:** culture 1x10^6^ cells/ well in 3 ml R10 medium + 200 U/ml GM-CSF

**Day 3:** Carefully remove about 1.5 ml of the medium from the top of the well and add same volume of fresh R10 medium with GM-CSF (NOTE: final concentration again 200 U/ml)

**Day 6:** Collect non-adherent cells as immature BMDC

Centrifuge at 485 x *g* for 5 minutes at 4°C and discard supernatant

Count cells and use for single cell staining

*Staining protocol of single cell suspensions:*

1. Use between 2-4 x 10^6^ cells per staining. Pellet cells by centrifugation at 485 x *g* for 5 minutes at 4°C in 5 ml polystyrene round bottom tubes.
2. Resuspend in 1 ml of Staining buffer to remove any residual Hank’s Salt Solution and centrifuge cells at 485 x *g* for 5 minutes at 4°C and discard supernatant
3. Resuspend cells in freshly prepared 100 µl Live/Dead dye solution
4. Incubate for 3 minutes at RT in the dark
5. Wash cells by adding 1 ml of Staining buffer to remove unbound viability dye, centrifuge tubes at 485 x *g* for 5 minutes at 4°C and discard supernatant
6. Resuspend cells in 100 µl of Fc block (diluted 1:100 - purified anti-mouse CD16/CD32, clone 217 2.4G2) in Staining Buffer
7. Incubate for 30 minutes at 4°C, protected from light
8. Wash cells by adding 1 ml of Staining buffer, centrifuge tubes at 485 x *g* for 5 minutes at 4°C and discard supernatant
9. Resuspend cells in 100 µl of antibody staining mix containing the correct final dilution of CCR7 and CCR2 antibodies in Brilliant Staining Buffer
10. Incubate for 30 minutes at 37°C in the dark
11. Wash cells by adding 1 ml of Staining buffer to remove unbound antibodies, centrifuge tubes at 485 x *g* for 5 minutes at 4°C and discard supernatant
12. Resuspend cells in 100 µl of antibody staining mix with the proper final dilutions of all antibodies for surface staining (and the required FMO mixes) diluted in Brilliant Staining Buffer.
13. For surface staining incubate for 30 minutes at 4°C, protected from light
14. Wash cells by adding 1 ml of PBS to remove unbound antibodies, centrifuge tubes at 485 x *g* for 5 minutes at 4°C and discard supernatant
15. Resuspend cells in 200 µl Fixation/Permeabilization Solution according to manufacturer’s instruction
16. Incubate for 15 minutes at 4°C protected from light
17. Wash cells by adding 500 µl Perm/Wash buffer 1X, centrifuge tubes at 485 x *g* for 5 minutes at 4°C and remove supernatant
18. Resuspend cells in 100 µl intracellular antibody mix containing CD207 (diluted in Perm/Wash buffer 1X) for intracellular staining
19. Incubate for 30 minutes at 4°C protected from light
20. Wash cells twice by adding 500 µl Perm/Wash buffer 1X, centrifuge tubes at 485 x *g* for 5 minutes at 4°C and remove supernatant
21. Resuspend cells in 100-200 µl of Perm/Wash buffer and keep in dark at 4°C until analysis on Cytek® Aurora

**Staining workflow for DC mouse panel**

|  | **Marker** | **Fluorochrome** | **Dilution** | **Diluent** | **Incubation (min/Temp)** | |
| --- | --- | --- | --- | --- | --- | --- |
| **1** | Viability dye e780 | APC-Cy7 | 1:5000 | PBS | | 3’/RT |
|  |  |  |  |  | |  |
| **2** | **Wash (Staining buffer)** | | | | | |
|  |  | | | | | |
| **3** | Fc Block | - | 1:100 | Staining Buffer | | 30’/4°C |
|  |  |  |  |  | |  |
| **4** | **Wash (Staining buffer)** | | | | | |
|  |  | | | | | |
| **5** | CCR7 | APC | 1:50 | Brilliant Stain Buffer | | 30’/37°C |
|  | CCR2 | BV711 | 1:400 |  | |  |
|  |  |  |  |  | |  |
| **6** | **Wash (Staining buffer)** | | | | | |
|  |  | | | | | |
| **7** | CD172a | FITC | 1:100 | Brilliant Stain Buffer | | 30’/4°C |
|  | NK1.1 | BB630 | 1:200 |  | |  |
|  | CD19 | BB660 | 1:400 |  | |  |
|  | CD40 | BB700 | 1:100 |  | |  |
|  | CD103 | BB790 | 1:200 |  | |  |
|  | F4/80 | PE-Dazzle594 | 1:100 |  | |  |
|  | CD3e | PE-Cy5 | 1:200 |  | |  |
|  | CD64 | PE-Cy7 | 1:200 |  | |  |
|  | PD-L2 | APC-R700 | 1:100 |  | |  |
|  | Ly-6C | BV421 | 1:200 |  | |  |
|  | MHC class II | BV480 | 1:400 |  | |  |
|  | Ly-6G | BV570 | 1:200 |  | |  |
|  | CD115 | BV605 | 1:100 |  | |  |
|  | XCR1 | BV650 | 1:400 |  | |  |
|  | pDCA-1 | BV750 | 1:400 |  | |  |
|  | CD24 | BV786 | 1:400 |  | |  |
|  | CD11c | BUV395 | 1:100 |  | |  |
|  | CD11b | BUV496 | 1:400 |  | |  |
|  | PD-L1 | BUV615 | 1:400 |  | |  |
|  | CD4 | BUV661 | 1:400 |  | |  |
|  | CD8a | BUV737 | 1:400 |  | |  |
|  | CD45 | BUV805 | 1:200 |  | |  |
|  |  |  |  |  | |  |
| **8** | **Wash (PBS)** | | | | | |
|  |  |  |  |  | |  |
| **9** | Fixation |  |  | Fixation/Permeabilization Solution | | 15’/4°C |
|  |  |  |  |  | |  |
| **10** | **Perm/Wash buffer 1X** | | | | | |
|  |  |  |  |  | |  |
| **11** | CD207 | A546 | 1:100 | Perm/Wash buffer | | 30’/4°C |
|  |  |  |  |  | |  |
| **12** | **Perm/Wash buffer 1X** | | | | | |
|  |  |  |  |  | |  |
| **13** | **Resuspend cells in 100-200 µl of Perm/Wash buffer and keep in dark at 4°C until acquisition  (max 2 hours)** | | | | | |

**Single Color Reference Control**

Single stains of each antibody with either cells or beads were prepared. For unmixing, splenocytes, day 6 bone marrow-derived DC (BMDC) and the VersaComp Antibody Capture Bead Kit were used, see table below. In our experience, using cells as single stained reference controls is superior to beads. Thus, we chose beads only in cases, when we could not separate positive and negative signals properly with the original antibody and the dummy approach (substitute with same fluorochrome from the same company conjugated to an antibody against an abundantly expressed marker) did not lead to satisfying results. Correct unmixing was controlled with single stained cells and adjusted manually if necessary.

**Supplementary Table 4: List of single stains used for unmixing of fluorochromes**

| Fluorochrome | Cells/Beads | Reference Control |
| --- | --- | --- |
| FITC | Splenocytes + unstained splenocytes (for negative gate) | CD45 FITC substitute |
| BB630 | Splenocytes | NK1.1 BB630P |
| BB660 | Splenocytes | CD19 BB660 |
| BB700 | Beads | CD40 BB700 |
| BB790 | Splenocytes | CD103 BB790 |
|  |  |  |
| A546 | Beads | CD207 A546 |
| PE-Dazzle594 | BMDC + unstained BMDC  (for negative gate) | F4/80 PE-Dazzle594 |
| PE-Cy5 | Splenocytes | CD3e PE-Cy5 |
| PE-Cy7 | BMDC | CD64 PE-Cy7 |
|  |  |  |
| APC | Splenocytes | CD3e APC substitute |
| APC-R700 | Beads | PD-L2 APC-R700 |
| APC-Cy7 | Splenocytes | Viability dye eF780 |
|  |  |  |
| BV421 | Splenocytes | Ly-6C BV421 |
| BV480 | Splenocytes | MHC-II BV480 |
| BV570 | Splenocytes | Ly-6G BV570 |
| BV605 | Splenocytes | CD24 BV605 substitute |
| BV650 | Splenocytes | CD4 BV650 substitute |
| BV711 | Beads | CCR2 BV711 |
| BV750 | Beads | pDCA-1 BV750 |
| BV786 | Splenocytes | CD24 BV786 |
|  |  |  |
| BUV395 | BMDC | CD11c BUV395 |
| BUV496 | BMDC + unstained BMDC  (for negative gate) | CD11b BUV496 |
| BUV615 | Beads | PD-L1 BUV615 |
| BUV661 | Splenocytes | CD4 BUV661 |
| BUV737 | Splenocytes | CD8 BUV737 |
| BUV805 | Splenocytes + unstained splenocytes (for negative gate) | CD45 BUV805 |

*Staining protocol for single color reference controls using cells*

1. Use up to 2x10^6^ cells per single stain. Pellet cells by centrifugation at 485 x *g* for 5 minutes at 4°C in 5 ml polystyrene round bottom tubes
2. Resuspend cells in 100 µl Staining buffer
3. Add the predetermined amount of antibody to the matching tube
4. Incubate for 30 minutes at 4°C, protected from light
5. Wash cells by adding 1 ml of PBS, centrifuge tubes at 485 x *g* for 5 minutes at 4°C and discard supernatant
6. Resuspend cells in 200 µl Fixation/Permeabilization Solution according to manufacturer’s instruction
7. Incubate for 15 minutes at 4°C protected from light
8. Wash cells by adding 500 µl Perm/Wash buffer 1X, centrifuge tubes at 485 x *g* for 5 minutes at 4°C and remove supernatant
9. Resuspend cells in 100-200 µl of Perm/Wash buffer and keep in dark at 4°C until acquisition
10. Collect an appropriate amount of events within your negative and positive gate of interest

*Staining protocol for single color reference controls using beads*

The VersaComp Antibody Capture Bead Kit (Beckman Coulter Life Sciences #B22804) contain VersaComp Antibody Capture Negative Beads and VersaComp Antibody Capture Positive Beads.

1. Bring Versa Comp beads to room temperature
2. Place 1 drop of negative beads and 1 drop of positive beads for each single color compensation control into 5 ml polystyrene round bottom tubes
3. Add 100 µl of PBS to each tube
4. Add the predetermined amount of antibody to the matching tube
5. Incubate for 30 minutes at 4°C protected from light
6. Wash beads by adding 1 ml PBS, centrifuge at 485 x *g* for 5 minutes at 4°C and discard supernatant
7. Resuspend beads in 200 µl Fixation/Permeabilization Solution according to manufacturer’s instruction
8. Incubate for 15 minutes at 4°C protected from light
9. Wash beads by adding 500 µl Perm/Wash buffer 1X, centrifuge tubes at 485 x *g* for 5 minutes at 4°C and remove supernatant
10. Resuspend beads in 100-200 µl of Perm/Wash buffer and keep in dark at 4°C until acquisition
11. Collect 10 000 events with at least 80% within the bead region of interest gate

**Titration of antibodies**

All antibodies were titrated, either to saturation or to a selected optimal concentration. Optimal antibody concentrations were considered as the lowest amount of antibody that exhibits the best signal separation with minimal background staining.


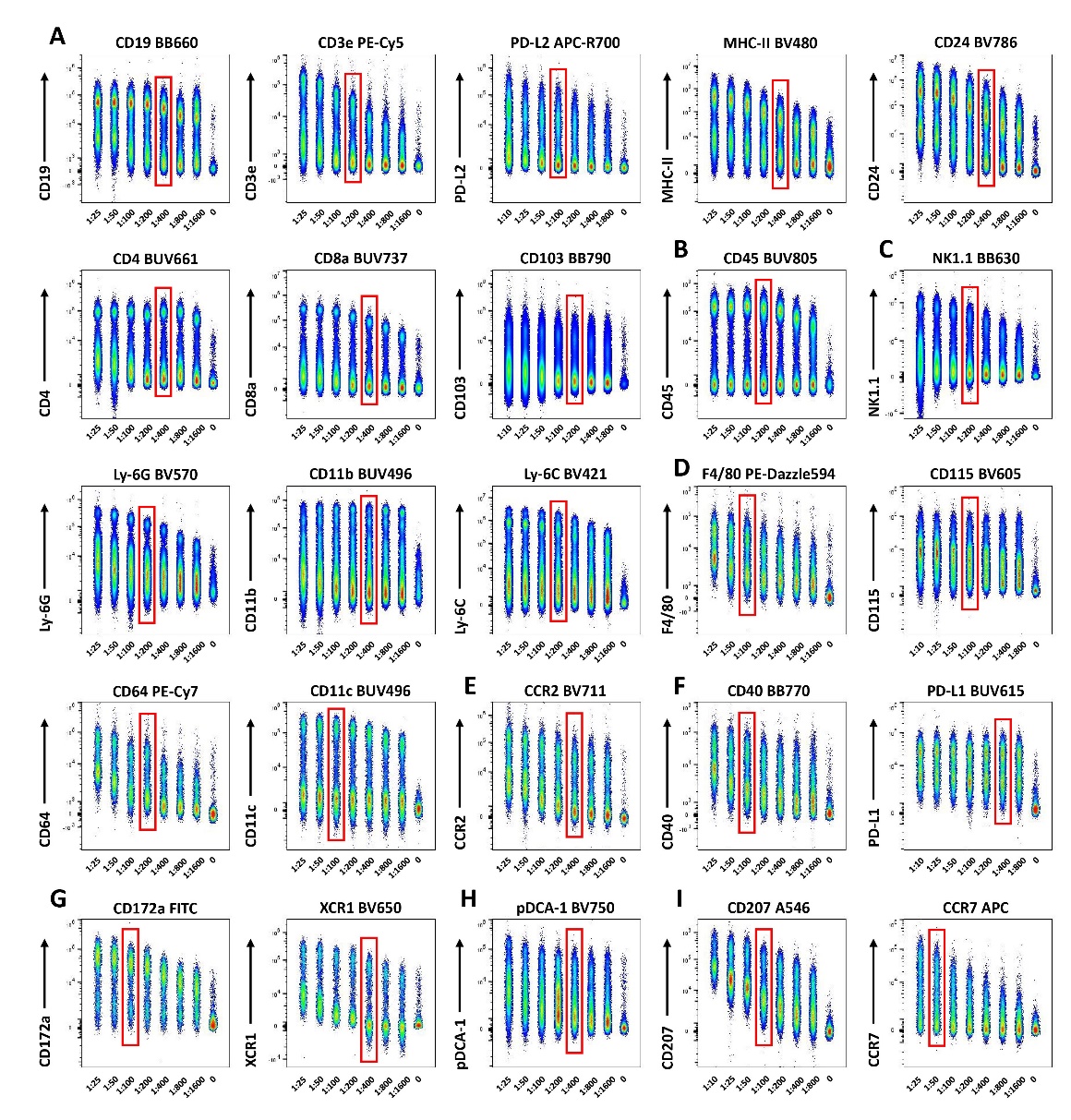


**Supplementary Figure S2. Antibody titrations for panel optimization:** Antibody titrations were performed using splenocytes and lymph node cells from 6-8 week old C57BL/6 mice, by performing at least seven 2-fold dilution steps (the exact dilutions are indicated on the x-axis). Unstained samples are indicated as 0 and displayed next to the lowest concentration of antibodies. For all titrations performed, non-specific Fc-receptor-mediated antibody binding was blocked with incubation for 15 minutes with anti-CD16/32 antibody (clone 2.4G2). After a washing step, cells were stained in a final volume of 100 µl and incubated for 30 minutes at 4°C, protected from light. Individual .fcs files were concatenated using FlowJo 10.7.1 for visualizing all dilutions in a single plot. The lowest amount of antibody that exhibits the best signal separation with minimal background staining was considered as the optimal antibody concentration. Antibody dilutions chosen for the optimized panel are highlighted in red **A.** Splenocytes **B.** Splenocytes and unstained splenocytes appended **C.** CD3^-^ CD19^-^ splenocytes **D.** CD11b^+^ splenocytes **E.** CD11b^int^ splenocytes **F.** CD11c^+^ splenocytes **G.** CD11c^+^ MHC-II^+^ splenocytes **H.** CD11c^int^ MHC-II^int^ splenocytes **I.** CD11c^+^ MHC-II^+^ lymph node cells

**Panel Development and Optimization Process**

The subsequent changes in the antibody panel during the optimization process are highlighted in yellow in Supplementary Table 5. The explanations for these alterations are described in detail in Supplementary Table 6. Some examples for these changes in panel design to develop the optimized panel are illustrated in Supplementary Figures S3, S4, and S5.

**Supplementary Table 5: Panel Iterations**

|  | **Iteration 1** | **Iteration 2** | **Iteration 3** | **Final panel** |
| --- | --- | --- | --- | --- |
| **FITC** | CD45 (30-F11) | Viability dye FVS520 | CD172a (P84) | CD172a (P84) |
| **BB630** | NK1.1 (PK136) | NK1.1 (PK136) | NK1.1 (PK136) | NK1.1 (PK136) |
| **BB660** | CD19 (1D3) | CD19 (1D3) | CD19 (1D3) | CD19 (1D3) |
| **BB700** | CD40 (3/23) | CD40 (3/23) | CD40 (3/23) | CD40 (3/23) |
| **BB790** | CD103 (M290) | CD103 (M290) | CD103 (M290) | CD103 (M290) |
|  |  |  |  |  |
| **PE/A546** | CD207 (L31) | CD207 (L31) | EpCam (G8.8) | CD207 (929F3.01) |
| **PE-CF594 / PE-Dazzle594** | CD3 (145-2C11) | Flt3 (A2F10.1) | F4/80 (BM8) | F4/80 (BM8) |
| **PE-Cy5** |  | CD3 (145-2C11) | CD3 (145-2C11) | CD3 (145-2C11) |
| **PE-Cy7** | CD64 (X54) | CD64 (X54) | CD64 (X54) | CD64 (X54) |
|  |  |  |  |  |
| **APC** | CCR7 (4B12) | CCR7 (4B12) | CCR7 (4B12) | CCR7 (4B12) |
| **APC-R700** | PD-L2 (TY25) | PD-L2 (TY25) | PD-L2 (TY25) | PD-L2 (TY25) |
| **APC-Cy7** | Ly-6C (HK1.4) | Ly-6C (HK1.4) | Viability dye eF780 | Viability dye eF780 |
|  |  |  |  |  |
| **BV421** | F4/80 (BM8) | F4/80 (BM8) | Ly-6C (HK1.4) | Ly-6C (HK1.4) |
| **BV480** | MHC class II (M5/114) | MHC class II (M5/114) | MHC class II (M5/114) | MHC class II (M5/114) |
| **BV570** | Ly-6G (1A8) | Ly-6G (1A8) | Ly-6G (1A8) | Ly-6G (1A8) |
| **BV605** | CD115 (T38-320) | CD115 (T38-320) | CD115 (T38-320) | CD115 (T38-320) |
| **BV650** | XCR1 (ZET) | XCR1 (ZET) | XCR1 (ZET) | XCR1 (ZET) |
| **BV711** | CCR2 (475301) | CCR2 (475301) | CCR2 (475301) | CCR2 (475301) |
| **BV750** | pDCA-1 (927) | pDCA-1 (927) | pDCA-1 (927) | pDCA-1 (927) |
| **BV786** |  | CD24 (M1/69) | CD24 (M1/69) | CD24 (M1/69) |
|  |  |  |  |  |
| **BUV395** | CD11c (N418) | CD11c (N418) | CD11c (N418) | CD11c (N418) |
| **BUV496** | CD11b (M1/70) | CD11b (M1/70) | CD11b (M1/70) | CD11b (M1/70) |
| **BUV615** | PD-L1 (MIH5) | PD-L1 (MIH5) | PD-L1 (MIH5) | PD-L1 (MIH5) |
| **BUV661** |  | CD4 (RM4-5) | CD4 (RM4-5) | CD4 (RM4-5) |
| **BUV737** | CD8a (53-6.7) | CD8a (53-6.7) | CD8a (53-6.7) | CD8a (53-6.7) |
| **BUV805** |  | CD45 (30-F11) | CD45 (30-F11) | CD45 (30-F11) |

**Supplementary Table 6: Explanations for panel iterations**

| **Specificity** | **Fluorochrome** | **Clone** | **Reason** |
| --- | --- | --- | --- |
| CD45 | FITC | 30-F11 | CD45 was switched to BUV805 to free up FITC |
| Vibility dye FVS520 | FITC | - | A fixable viability stain was added to exclude dead cells from flow cytometry analysis. |
| CD3 | PE-CF594 | 145-2C11 | CD3 was switched to PE-Cy5. This fluorochrome was initially not used, as the spillover into other channels is considerable. However, as we focus on DC in this panel and CD3 is not expressed on any other cells than T cells, high similarity values are not of concern. |
| Flt3 | PE-CF594 | A2F10.1 | Flt3 was included to evaluate if it is an additional helpful marker to discriminate DC (pan DC marker). Optimization process revealed that Flt3 did not serve as a pan DC marker. Furthermore, it was not useful for the separation of different DC subsets. Therefore, this marker was removed from the panel. |
| CD24 | BV786 | M1/69 | Included to allow a better separation of cDC1 and LC |
| CD4 | BUV661 | RM4-5 | Included to allow further analysis of CD3^+^ T cells |
| CD172a | FITC | P84 | CD172a was included to subdivide cDC2 and LC from XCR1^+^ expressing cDC1 |
| Viability dye eF780 | APC-Cy7 | - | The fixable viability dye was switched to the APC-Cy7 channel to free up FITC. New included CD172a FITC is not essential for DC subset discrimination, therefore the FITC cannel can be used for reporter mice expressing GFP. |
| CD207 | PE | L31 | Was excluded, as the L31 clone gave no signal in lymph node tissue and we changed to EpCam to identify LC (see Online Fig. 4). |
| EpCam | PE | G8.8 | Allows the identification of LC in combination with CD24 (see Online Fig. 4). EpCam was removed and replaced by a different clone of CD207 (929F3.01) (intracellular staining) |
| CD207 | A546 | 929F3.01 | CD207, clone 929F3.01 (intracellular staining) was used instead of EpCam to better discriminate LC |
| Ly-6C | APC-Cy7 | HK1.4 | Ly-6C caused a lot of spreading error into different channels, especially the PE-Cy7 was affected, resulting in an unsatisfying resolution of the CD64 signal. Ly-6C was therefore moved to BV421. |
| F4/80 | BV421 | BM8 | Poor separation of positive and negative signal. F4/80 was moved to PE-Dazzle594 (see Online Fig. 5). |

**Orange filling**: new included reagents

**Green filling**: reagents that were excluded

**Blue filling**: reagents which have changed fluorochrome

**Grey filling**: reagents that were included for panel optimization but were excluded for the final panel

As we realized during the optimization process that staining the chemokine receptors CCR7 and CCR2 at 4°C gave no signal, we improved chemokine receptor staining by incubation at 37°C (Supplementary Fig. S3 and S4).

**Supplementary Figure 3**
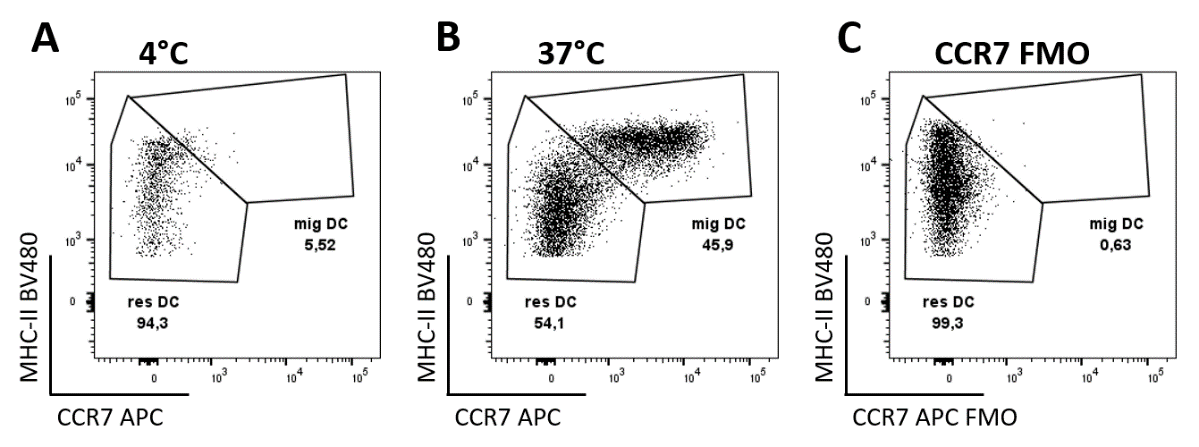


**Supplementary Figure S3: Comparison of CCR7 staining in lymph node cells for panel optimization.** Lymph node tissue from C57BL/6 mice was used and the full panel was tested, to identify best staining conditions for CCR7. After excluding dead cells, B cells, NK cells, T cells, pDC, monocytes and neutrophils, the remaining CD11c^+^ cells were used to identify DC (same gating as in Fig. 1). **A.** CCR7 APC antibody was incubated for 30 minutes at 4°C together with all the other surface markers. **B.** CCR7 APC antibody was separately incubated for 30 minutes at 37°C, before staining the remaining surface molecules at 4°C. **C.** CCR7 APC FMO stain control. mig DC: migratory DC, res DC: resident DC

**Supplementary Figure 4**


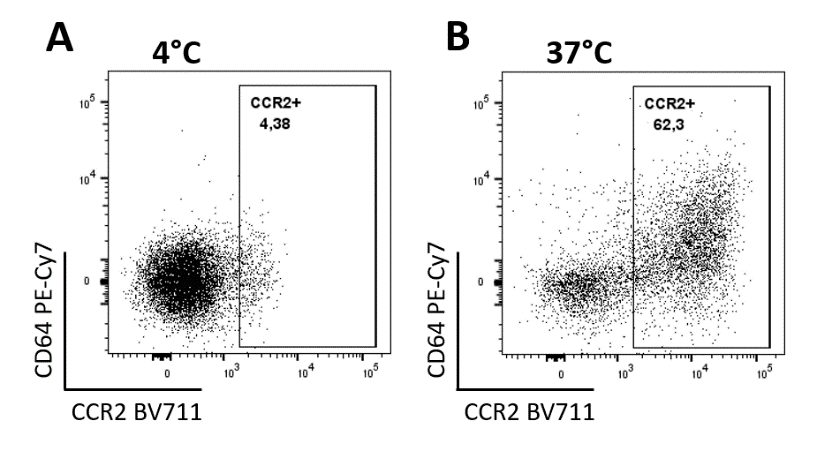


**Supplementary Figure S4: Comparison of CCR2 staining in lymph node cells for panel optimization**. Lymph node tissue from C57BL/6 mice was used and the full panel was tested, to identify best staining conditions for the chemokine receptor CCR2. After excluding dead cells, B cells, NK cells, T cells and pDC, CCR2 staining was analyzed on Ly-6C expressing monocytes (same gating as in Fig. 1). **A**. CCR2 BV711 antibody was incubated for 30 minutes at 4°C together with all the other surface markers. **B**. CCR2 BV711 antibody was separately incubated for 30 minutes at 37°C, before staining the remaining surface molecules at 4°C. Note that after the 37°C incubation step with the CCR2 and CCR7 antibody, subsequent staining for surface marker CD64 is improved. This is most likely due to enzymatic cleavage of these molecules during cell isolation process with re-expression on surface during the 37° incubation.

We also tested different approaches for identification of LC by antibodies against EpCam or Langerin (Supplementary Fig. S5). Langerin is quickly internalized during LC migration [4], so the clone L31 staining extracellular Langerin was exchanged with EpCam after showing no signal [5]. However, EpCam staining was not very discriminatory in LN, so we decided to use the antibody clone 929F3.01 that detects an intracellular epitope of Langerin [6] and gave the best discrimination of LC (Supplementary Fig. S5).

**Supplementary Figure 5**


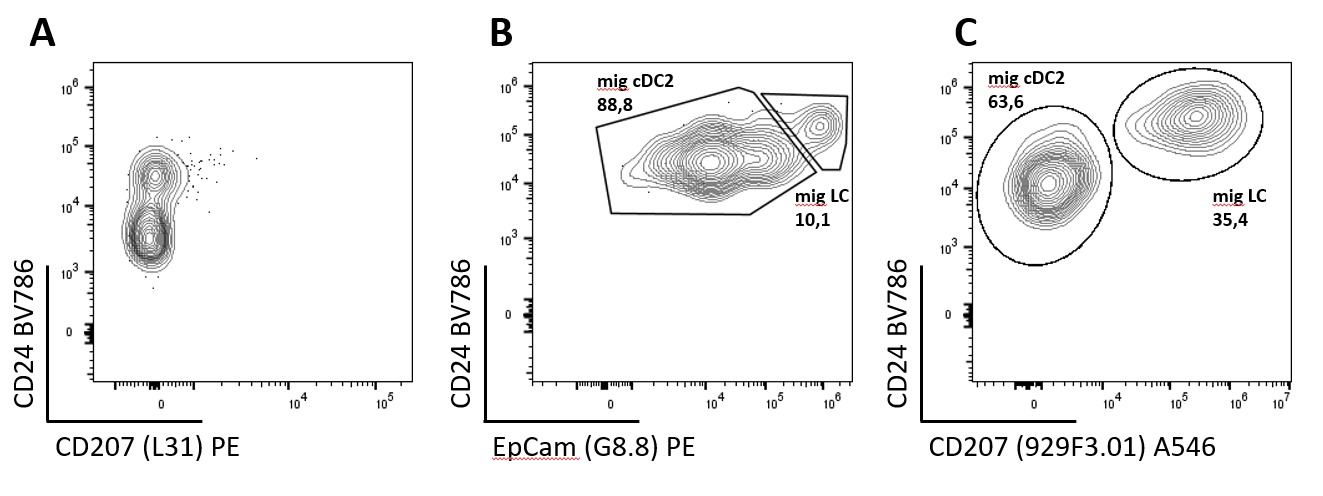


**Supplementary Figure S5: Comparison of different antibodies and clones to gate LC for panel optimization.** Skin-draining lymph nodes from C57BL/6 mice were isolated and the full panel was stained to analyze the best way to identify LC. After excluding dead cells, B cells, NK cells, T cells, pDC, monocytes and neutrophils, the remaining CD11c^+^ cells were used to identify DC. Different antibodies and clones were analyzed on migratory CD172a^+^ cells (same gating as in Fig. 1). **A.** CD207 (clone L31) was used to gate LC. **B.** EpCam (clone G8.8) was suboptimal to identify LC. **C.** Intracellular staining using CD207 (clone 929F3.01) was optimal to identify LC. mig cDC2: migratory cDC2, mig LC: migratory LC. The signal intensity of CD24 BV786 differs between the different plots as these flow cytometry analysis were performed in separate panel iteration experiments.

**Supplementary Figure 6**

**
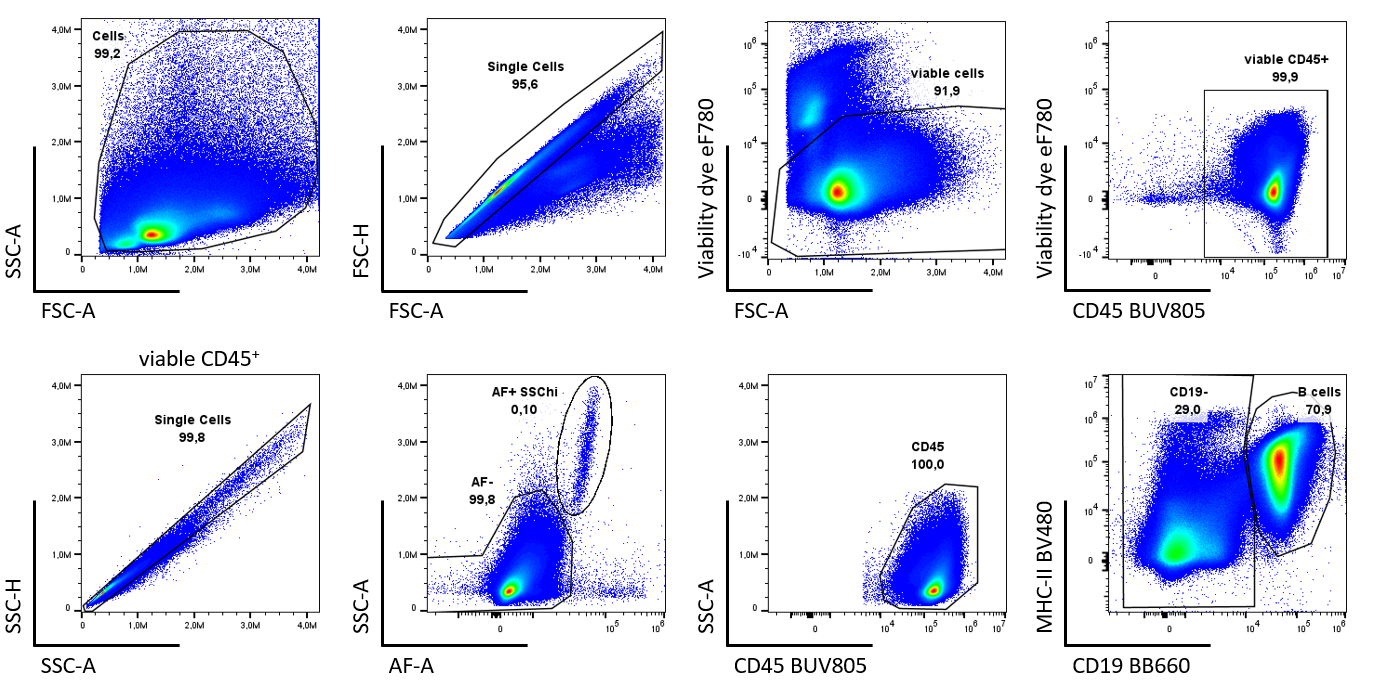
**

**Supplementary Figure S6. Several preclearance steps were performed.** Cellular debris, doublets and dead cells were removed. After gating on viable CD45^+^ cells, single cell gate was repeated using the side scatter instead of the forward scatter, and autofluorescent cells were removed. AF: Autofluorescence.

**Supplementary Figure 7**


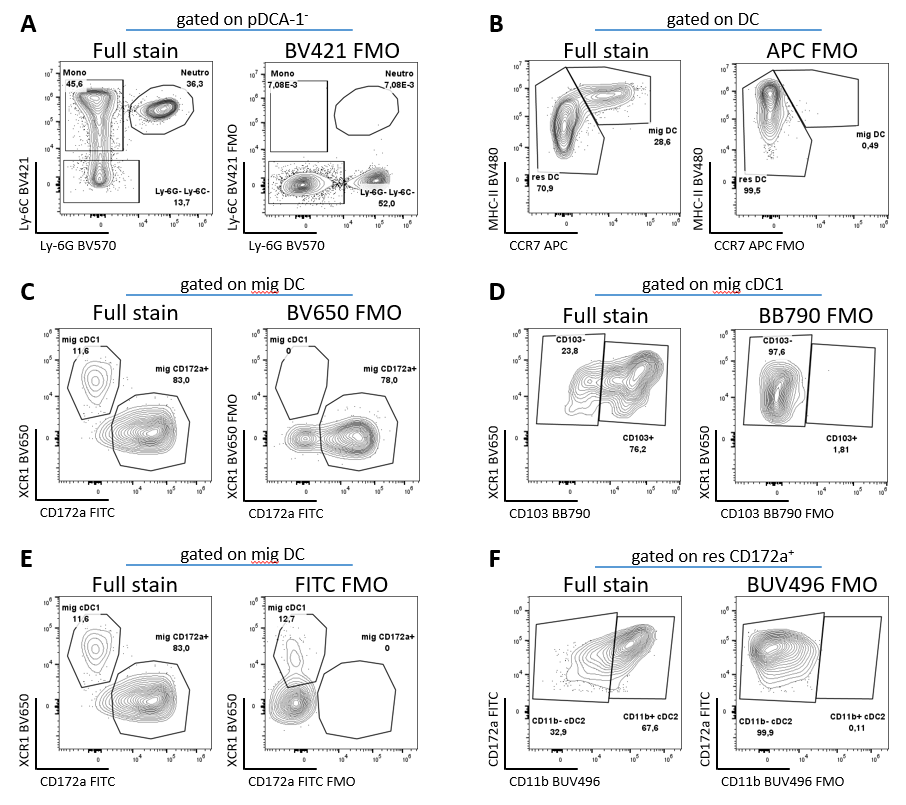


**Supplementary Figure S7: Selected fluorescent minus one (FMO) staining controls for optimizing the panel for skin-draining lymph node cells.** FMO controls were performed using the same gating as in main Fig. 1. A. BV421 FMO to better discriminate monocytes and neutrophils. B. APC FMO allows a better separation of migratory and resident DC. C. BV650 FMO to aid in the identification of XCR1 expressing migratory cDC1. D. BB790 FMO to identify CD103^+^ cDC1. E. FITC FMO for defining CD172a^+^ subset more accurate. F. BUV496 FMO to assist in CD11b^+^ cDC2 subset delineation. Mono: monocytes, Neutro: neutrophils, mig DC: migratory DC, res DC: resident DC

**Supplementary Figure 8**


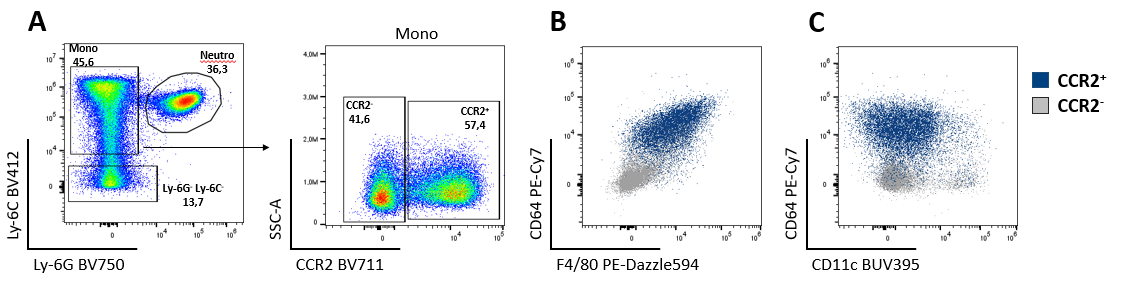


**Supplementary Figure S8: Analysis of Ly-6C^+^ monocytes. A.** After removing dead cells, B cells, NK cells, T cells and pDC, Ly-6C expressing monocytes were gated. Monocytes can be distinguished in CCR2^+^ and CCR2^-^ cells. **B.** Cells that are expressing the chemokine receptor CCR2 are mainly positive for the markers F4/80 and CD64, indicating that these are recruited monocytes. CCR2^-^ monocytic cells are lacking the expression of these two markers. **C.** Very few cells are CD11c positive monocyte-derived DC (moDC) which are characterized by their expression of Ly-6C, CD64 and CD11c. This is not surprising due to the fact that we are showing flow cytometry analysis of lymph nodes of healthy mice, where recruitment of inflammatory moDC is not to be expected. Mono: monocytes, Neutro: neutrophils

**UMAP Analysis for lymph nodes**

Analysing these high-dimensional single-cell datasets with traditional manual two-dimensional gating is difficult and time consuming. Furthermore, as in practice not all combinations of markers are examined, valuable information on cell populations might remain unexplored and undetected. Several computational techniques have been described in the last years to analyze and visualize multiparameter flow data [7], including dimensionality reduction algorithms, like t-stochastic neighbour embedding (t-SNE) and uniform manifold approximation projection (UMAP) or unsupervised clustering methods like FlowSOM [8, 9]. A representative UMAP analysis and visualization of the different DC subsets is shown in Supplementary Figure S8 [10]. For example, PD-L1, CD40 and CCR7 expression is high on migratory DC subsets similarly to Fig.1G. The UMAP analysis allows to visualize higher PD-L2 levels on migratory cDC2 and cDC1, whereas LC show lower ones. Note that some monocyte markers, such as CD64, CCR2 and F4/80 are also expressed by resident and migratory cDC2 which could be due to either upregulation of these markers by DC or due to a contamination by monocyte-derived cells, such as monocyte-derived DC (moDC) [11].


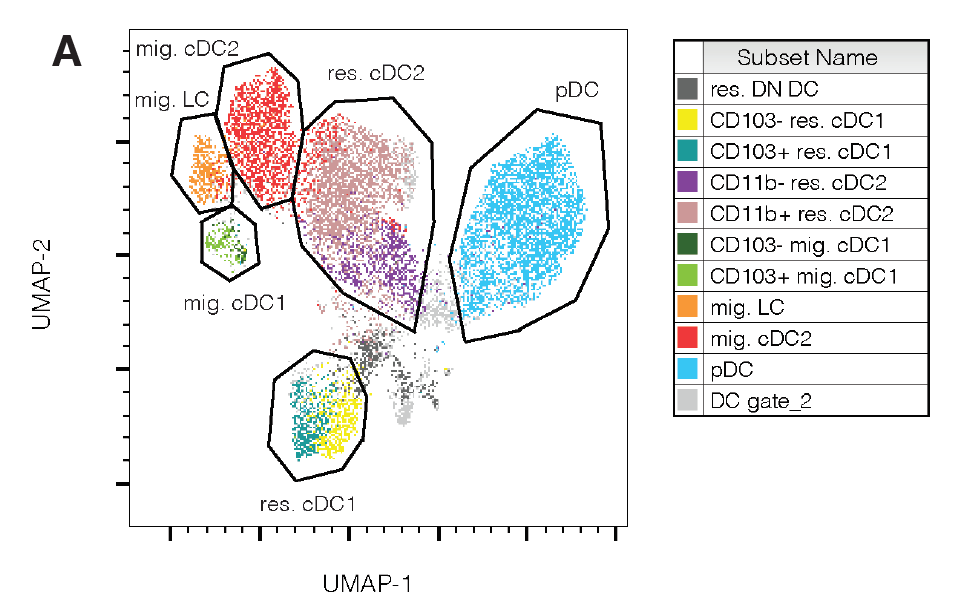

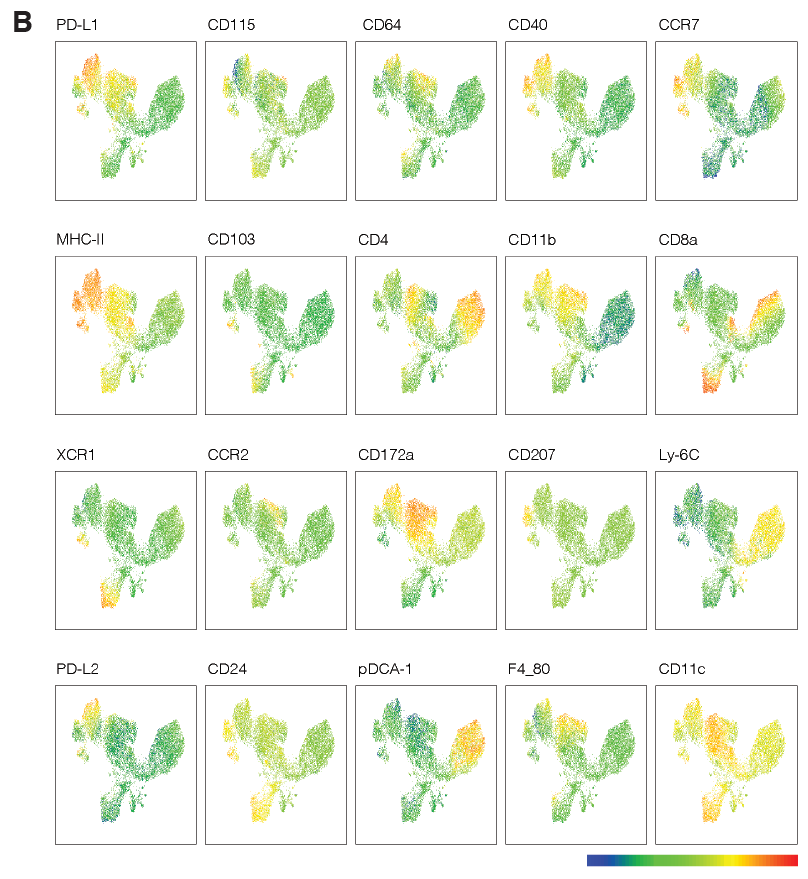


**Supplementary Figure S9: UMAP analysis of the final 26-color flow cytometry panel on mouse skin-draining lymph node cells.** For the dimensionality reduction approach we excluded B cells, T cells and NK cells from the analysis and defined DC by their expression of CD11c and MHC-II. Uniform Manifold Approximation and Projection (UMAP) was applied on the DC gate using a plugin integrated in FlowJo software. **A.** Overlays with manual gated populations. **B.** Expression levels of different markers.

**Supplementary Figure 10**

**
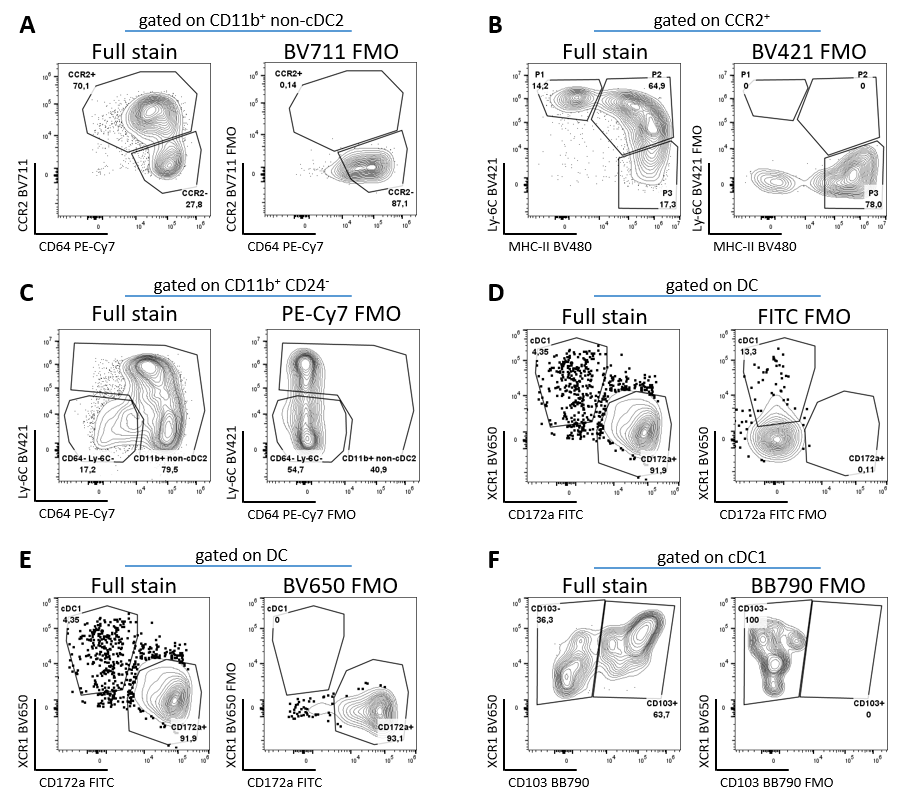
**

**Supplementary Figure S10: Selected fluorescent minus one (FMO) staining controls for optimizing the panel for mouse ear skin.** FMO controls were performed using the same gating as in Online Fig. 11.  **A.** BV711 FMO to distinguish between CCR2^+^ and CCR2^-^ cells. **B.** BV421 FMO for a better discrimination of different dermal monocytes and moDC subpopulations. **C.** PE-Cy7 FMO. **D.** FITC FMO to assist in CD172a^+^ subset delineation **E.** BV650 FMO for better separation of XCR1^+^ cDC1. **F.** BB790 FMO to identify CD103^+^ cDC1.

**UMAP Analysis for skin**

As already mentioned above analysing these high-dimensional single-cell datasets with traditional manual two-dimensional gating is difficult and time consuming. We here now also show a representative UMAP analysis and visualization of the different DC subsets and non-DC populations (P1-P5) in the skin as shown in Supplementary Figure S10. This analysis shows that CD64 expression is higher on CCR2^-^ cells (P4 and P5) compared to non-DC population P1, P2 and P3, similar to Fig. 2. Furthermore, the UMAP analysis demonstrates that LCs are also expressing F4/80 [12].


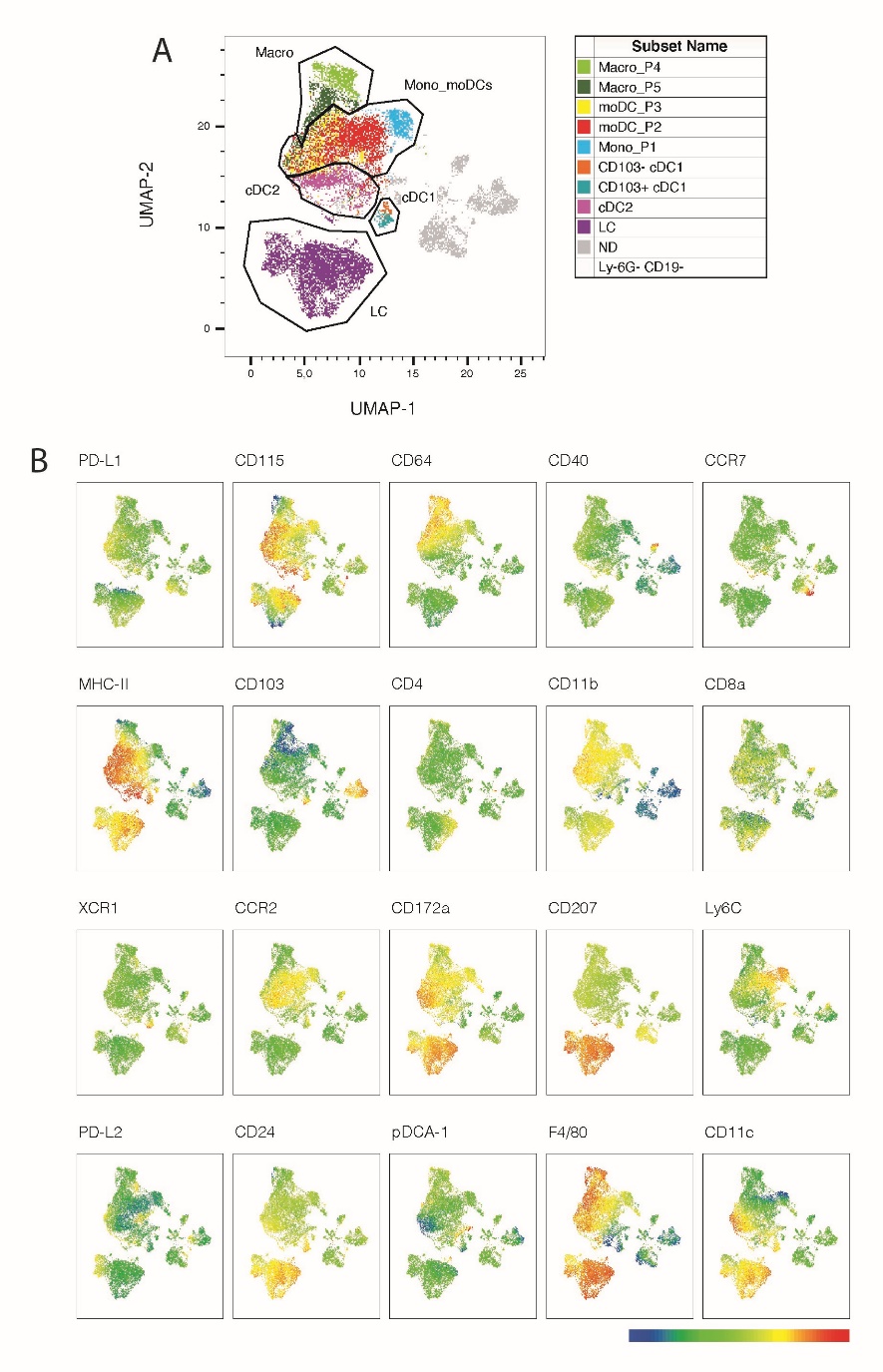


**Supplementary Figure S11: UMAP analysis of the final 26-color flow cytometry panel on mouse ear skin.** For the dimensionality reduction approach, dead cells, NK cells, T cells, B cells and neutrophils were excluded. Uniform Manifold Approximation and Projection (UMAP) was applied on the Ly-6G^-^ CD19^-^ gate (Online Fig. 11B) using a plugin integrated in FlowJo software. **A.** Overlays with manual gated populations. **B.** Expression levels of several different markers. P1: Ly-6C^high^ MHC-II^-^ dermal monocytes, P2: Ly-6C^high^ MHC-II^+^ dermal moDC, P3: Ly-6C^-^ MHC-II^+^ dermal moDC, P4: Ly-6C^-^ MHC-II^-^ dermal macrophages, P5: Ly-6C^-^ MHC-II^+^ dermal macrophages, LC: Langerhans cells, ND: not defined

In summary, this novel 26-color flow cytometry panel was optimized for the phenotyping of myeloid subsets with a special focus on DC in healthy LN and skin. In addition to resolving the complexity of myeloid cells, this panel also provides basic information on the main lymphoid subsets. For future use, several adaptions of the panel are possible, e.g. to use a viability dye excited by the UV laser (FVS 440UV) or to free the FITC channel for reporter mice expressing GFP as CD172a/SIRPα is not essential for DC subset discrimination. Moreover, if LC are not present in the tissue of interest, the Langerin antibody can be substituted by other intracellular markers. How well this panel works for diseased organs needs to be verified in future as myeloid subset marker expression might be altered due to inflammation, infection or tumor microenvironment. In addition, different digestion protocols could affect the availability of surface markers and antibody binding. However, since we used already two different proteases, namely collagenase and liberase to establish this panel, we are convinced that it will be applicable for most applications. We believe that this panel can be used as a well-designed backbone for extended flow cytometry panels and easily adapted for specific research questions in the field of mouse myeloid immunology.

**References**

1 **Guilliams, M., Dutertre, C. A., Scott, C. L., McGovern, N., Sichien, D., Chakarov, S., Van Gassen, S., Chen, J., Poidinger, M., De Prijck, S., Tavernier, S. J., Low, I., Irac, S. E., Mattar, C. N., Sumatoh, H. R., Low, G. H., Chung, T. J., Chan, D. K., Tan, K. K., Hon, T. L., Fossum, E., Bogen, B., Choolani, M., Chan, J. K., Larbi, A., Luche, H., Henri, S., Saeys, Y., Newell, E. W., Lambrecht, B. N., Malissen, B. and Ginhoux, F.,** Unsupervised High-Dimensional Analysis Aligns Dendritic Cells across Tissues and Species. *Immunity* 2016. **45**: 669-684.

2 **Malissen, B., Tamoutounour, S. and Henri, S.,** The origins and functions of dendritic cells and macrophages in the skin. *Nat Rev Immunol* 2014. **14**: 417-428.

3 **Nolan, J. P. and Condello, D.,** Spectral flow cytometry. *Curr Protoc Cytom* 2013. **Chapter 1**: Unit1 27.

4 **Douillard, P., Stoitzner, P., Tripp, C. H., Clair-Moninot, V., Ait-Yahia, S., McLellan, A. D., Eggert, A., Romani, N. and Saeland, S.,** Mouse lymphoid tissue contains distinct subsets of langerin/CD207 dendritic cells, only one of which represents epidermal-derived Langerhans cells. *The Journal of investigative dermatology* 2005. **125**: 983-994.

5 **Nagao, K., Ginhoux, F., Leitner, W. W., Motegi, S., Bennett, C. L., Clausen, B. E., Merad, M. and Udey, M. C.,** Murine epidermal Langerhans cells and langerin-expressing dermal dendritic cells are unrelated and exhibit distinct functions. *Proc Natl Acad Sci U S A* 2009. **106**: 3312-3317.

6 **Stoitzner, P., Holzmann, S., McLellan, A. D., Ivarsson, L., Stossel, H., Kapp, M., Kammerer, U., Douillard, P., Kampgen, E., Koch, F., Saeland, S. and Romani, N.,** Visualization and characterization of migratory Langerhans cells in murine skin and lymph nodes by antibodies against Langerin/CD207. *J Invest Dermatol* 2003. **120**: 266-274.

7 **Saeys, Y., Van Gassen, S. and Lambrecht, B. N.,** Computational flow cytometry: helping to make sense of high-dimensional immunology data. *Nat Rev Immunol* 2016. **16**: 449-462.

8 **Van Gassen, S., Callebaut, B., Van Helden, M. J., Lambrecht, B. N., Demeester, P., Dhaene, T. and Saeys, Y.,** FlowSOM: Using self-organizing maps for visualization and interpretation of cytometry data. *Cytometry A* 2015. **87**: 636-645.

9 **Weber, L. M. and Robinson, M. D.,** Comparison of clustering methods for high-dimensional single-cell flow and mass cytometry data. *Cytometry A* 2016. **89**: 1084-1096.

10 **Becht, E., McInnes, L., Healy, J., Dutertre, C. A., Kwok, I. W. H., Ng, L. G., Ginhoux, F. and Newell, E. W.,** Dimensionality reduction for visualizing single-cell data using UMAP. *Nat Biotechnol* 2018.

11 **Langlet, C., Tamoutounour, S., Henri, S., Luche, H., Ardouin, L., Gregoire, C., Malissen, B. and Guilliams, M.,** CD64 expression distinguishes monocyte-derived and conventional dendritic cells and reveals their distinct role during intramuscular immunization. *J Immunol* 2012. **188**: 1751-1760.

12 **Schuler, G. and Steinman, R. M.,** Murine epidermal Langerhans cells mature into potent immunostimulatory dendritic cells in vitro. *J Exp Med* 1985. **161**: 526-546.
